# Letter to the editor: Excess all-cause mortality during second wave of COVID-19 – the Polish perspective

**DOI:** 10.2807/1560-7917.ES.2021.26.7.2100117

**Published:** 2021-02-18

**Authors:** Jakub Grabowski, Natalia Witkowska, Leszek Bidzan

**Affiliations:** 1 Department of Developmental Psychiatry, Psychotic and Geriatric Disorders Faculty of Medicine, Medical University of Gdańsk, Poland; 2 Adult Psychiatry Scientific Circle, Department of Developmental Psychiatry, Psychotic and Geriatric Disorders, Faculty of Medicine, Medical University of Gdańsk, Poland

**Keywords:** epidemiology, public health, Europe, COVID-19

**To the editor:** Having read Nørgaard et al.’s article [1] about excess mortality during the second wave of the coronavirus disease (COVID-19) pandemic, we consider that no discussion on excess mortality during this period would be complete without mentioning some other highly affected European countries.

Bulgaria, Czech Republic, Poland and Romania are not part of the European monitoring of excess mortality for public health action (EuroMOMO) network, which means that their data are not included in the periodic z-score reports. However, Eurostat information for November 2020 [2,3] revealed that these four countries, with a total population of 75 million, were among the seven with highest excess mortality in the European Union (EU). The increase in the number of deaths for all causes, compared with 2016–2019, exceeded 50% in Romania, 75% in the Czech Republic and 90% in Bulgaria. Poland, with a 97% increase, had the highest excess mortality among all EU countries in November 2020. Other countries with over 50% increase include Belgium, Hungary and Slovenia, which means that six of seven countries with highest excess mortality lie in Central and Eastern Europe. In September 2020, Poland followed the Czech Republic (45% vs 53% increase). The Eurostat overview for December 2020 shows lower rates of excess mortality but confirms the discrepancies between different areas in Europe [4]. 

Furthermore, recently published Polish government statistical data for 2020 show that in this country with 38 million inhabitants, 79,000 excess deaths were reported, compared with 2019 [5]. In terms of mortality, this makes 2020 the worst year in Poland in over seven decades (12.4 deaths / 1,000 residents in 2020 vs 12.2 in 1951, second worst year in post-World War II history). It is estimated that only 28,500 of the 2020 deaths can be directly attributed to COVID-19, i.e. infection as a main reason of death or comorbidity, with the remaining 50,000 being associated with other causes [6].

These figures are disturbing when we take into consideration that case numbers and fatalities were relatively low during the first wave in Poland. The threshold of 1,000 deaths attributable to COVID-19 was reached only on 25 May 2020, compared to 12 March in Italy and 23 March in Spain [6].

A possible explanation of the rate of excess all-cause mortality in Poland during the second wave may involve several factors. Limited testing capacities were a problem in many countries during the first wave. A high rate of positive severe acute respiratory syndrome coronavirus 2 (SARS-CoV-2) tests, exceeding 20% in Poland in most of the discussed period with a peak of 50.3% on 24 November 2020 [6], suggests that COVID-19 was underdiagnosed. Consequently, some patients may have died because of COVID-19 and its complications before diagnosis was made. Furthermore, focusing on COVID-19 cases, many health professionals had to abandon their previous obligations, while telemedicine was introduced on a large scale with serious limitations of direct patient-doctor contact. Underdiagnosis of COVID-19 cases also meant that some pauci-symptomatic patients or people with only mild symptoms were not tested routinely, with their infection being confirmed only during screening in the emergency units of general hospitals, where they were admitted because of deterioration of COVID-19 or other somatic conditions. Diagnosis of COVID-19 in hospitalised patients impacted majorly on operations, as it led in several instances to quarantine of personnel and even closures of whole wards, limiting access to health services for the most severely ill people. Of note, health expenditure in Poland is low compared to other EU countries (Poland: 1,507 EUR per capita; EU average: 2,775 EUR per capita) and the number of health professionals per 1,000 inhabitants ranges at the lower end [7].

With the currently observed massive strain on the healthcare system, actions are necessary to address health issues other than COVID-19 during the ongoing pandemic. For example, sustaining efficient oncological diagnostics (7% decrease of fast-track oncological cards in Poland in 2020 vs. 2019) [8], cardiological services (15% decrease of myocardial infarction hospitalisations in Greater Poland region in 2020 vs. 2019) [9], brain stroke management (25% decrease in stroke patients treated with mechanical thrombectomy in Lesser Poland region in January–May 2020 vs. January–May 2019) [10] and managing an observed deterioration of the population’s mental health [11] are expected to be major challenges for the following months of the ongoing pandemic.
